# Individual Factors Associated with Health-Related Quality of Life in Facial Cutaneous Squamous Cell Carcinoma—A Pilot Study

**DOI:** 10.3390/cmtr19030037

**Published:** 2026-08-25

**Authors:** Oliver Wagendorf, Forogh Miethe, Jonas Wüster, Meikel Vesper, Steffen Koerdt, Carsten Rendenbach, Max Heiland, Susanne Nahles, Norbert Neckel

**Affiliations:** 1 Charité—Universitätsmedizin Berlin, Corporate Member of Freie Universität Berlin and Humboldt-Universität zu Berlin, Department of Oral and Maxillofacial Surgery, Hindenburgdamm 30, 12203 Berlin, Germany; 2 Department of Oral and Maxillofacial Surgery, Klinikum Barnim GmbH, Werner-Forßmann-Krankenhaus, 16225 Eberswalde, Germany; 3 Department of Oral and Maxillofacial Surgery, University Medical Center Freiburg, Faculty of Medicine, Albert Ludwig University of Freiburg, 79106 Freiburg im Breisgau, Germany

**Keywords:** cSCC, HR-QoL, surgical treatment, face

## Abstract

Purpose: Facial expressions and aesthetics are vital for communication, self-expression, and social interaction. Therefore, this study aimed to evaluate the impact of localization and size of facial cutaneous squamous cell carcinoma (cSCC) on changes in perceived pre- and postoperative health-related quality of life (HR-QoL). Methods: This retrospective study included patients who had undergone surgery for facial cSCC. Size, localization and stage were assessed. Pre- and postoperative HR-QoL were evaluated using the Skindex-29. Statistical analyses, including descriptive statistics, the X2 Test, Wilcoxon Test, and repeated-measures ANOVA, were performed to analyze demographic data, tumor characteristics, and changes in HR-QoL. Results: Forty patients (mean age of 78.5 years) were included. Postoperatively, significant improvements were observed in the overall, emotional, and symptomatic HR-QoL. Tumor dimensions had an impact on presurgical emotional distress. For tumor sizes from 6 to 15 mm, significant release in symptom burden and improvement in general HR-QoL were observed. While women experienced notable improvement only in emotional distress, men also exhibited improvement in symptoms, and overall HR-QoL. All age groups showed improvements in emotional distress, symptoms and overall HR-QoL. No significant differences were found in terms of age or localization. Conclusions: This study suggests that sex, tumor localization, and tumor size may interact in influencing postoperative HR-QoL outcomes in patients with cSCC. While surgical treatment by experienced specialists was associated with an overall improvement in HR-QoL following successful tumor eradication, these findings should be interpreted with caution given the limited sample size. The results indicate that surgery remains an important first-line treatment option, particularly for medium-sized tumors. In this cohort, TNM classification was not associated with postoperative HR-QoL changes; however, interpretation is limited by the predominance of early-stage tumors. Further studies with larger patient populations are required to confirm these observations and to better elucidate the relationship between clinical staging and HR-QoL outcomes.

## 1. Introduction

The human brain is highly specialized in processing facial features, allowing for the rapid extraction of information such as age, gender, and health status [1,2]. Even minor alterations in facial appearance can significantly impact social interactions and self-perception [1,2,3,4]. Malignant skin lesions in the facial region are particularly relevant in this context, as they often affect both aesthetic and functional aspects due to tumor infiltration or necessary therapeutic interventions [5].

Lately, the incidence of non-melanoma skin cancer (NMSC) has been increasing worldwide, with cutaneous squamous cell carcinoma (cSCC) being the second-most common type after basal cell carcinoma [6,7].

In Europe, the age-standardized incidence of cSCC varies between 35 and 108 per 100,000 individuals per year, depending on gender and region [8]. Key risk factors include cumulative sun exposure, fair skin, immunosuppression, and certain genetic predispositions such as xeroderma pigmentosum [9]. Given the high level of cumulative sun exposure, the facial skin is particularly affected, with approximately up to 80% of cSCC cases occurring in this region [10,11].

The primary treatment for localized cSCC is surgical excision with histological margin control, which enables high cure rates [5,12]. Alternative therapies such as radiotherapy and cryotherapy may be considered in inoperable cases or those with an elevated risk of recurrence [5,12]. For relatively small defects, tension-free primary closure is favored, ensuring optimal healing and minimal scarring [13,14]. In medium-sized defects, local flaps, including transposition, advancement, and rotation types, allow reconstruction with tissues similar in color and texture, thereby enhancing cosmetic outcomes while maintaining functional integrity [15]. However, beyond oncologic control, facial tumor resection also has implications for patient well-being, as both the disease and its treatment can cause visible alterations that affect health-related quality of life (HR-QoL) [16,17,18,19].

HR-QoL is a multidimensional concept that encompasses physical, psychological, and social well-being. Facial cSCC and its treatment impact HR-QoL by affecting self-image, self-esteem, and social interactions, potentially causing emotional distress [20]. HR-QoL, a key outcome measure in clinical research, is commonly assessed using the Skindex-29 [21,22,23,24,25,26,27,28,29]. Personalized treatment should balance oncologic control with HR-QoL considerations [22,23,24,25,26,27,28,29].

Given the visibility and psychological impact of facial appearance, tumor localization may influence HR-QoL changes [16,30]. Observers focus on the central facial triangle, considering tumors in highly visible areas (e.g., nose, lips) to be more distressing than those in less visible sites (e.g., earlobe) [31]. However, the specific impact of tumor localization on HR-QoL remains unclear, requiring further study.

To the best of our knowledge, existing studies on cSCC have primarily focused on oncologic outcomes or overall postoperative quality of life, without systematically analyzing the influence of tumor localization within the facial region. As a result, potential differences in HR-QoL changes related to functionally or aesthetically sensitive facial subunits remain insufficiently characterized. Therefore, the primary aim of this study was to evaluate changes in health-related quality of life (HR-QoL) from before to after surgical treatment of facial cutaneous squamous cell carcinoma (cSCC). Particular emphasis was placed on whether these changes were associated with tumor localization and tumor size, while potential associations with sex and age were assessed as secondary objectives.

## 2. Methods

### 2.1. Ethical Approval

The research proposal for this retrospective study was approved by the Ethics Committee of Charité Universitätsmedizin Berlin, Germany (EA2/177/21) and Landesärztekammer Brandenburg, Germany (2025-142-BO), and complied with the STROBE guidelines. The study conformed to the Declaration of Helsinki and the European Medicines Agency Guidelines for Good Clinical Practice. Informed consent was obtained from all participants. The study was retrospectively registered in the German Clinical Trials Register (DRKS00039394) after initiation of recruitment on 26 March 2026.

### 2.2. Inclusion Criteria

The study’s inclusion criteria were age of majority (18 years or older) and an initial diagnosis of cSCC of the facial skin.

### 2.3. Data Collection

**Figure 1:**
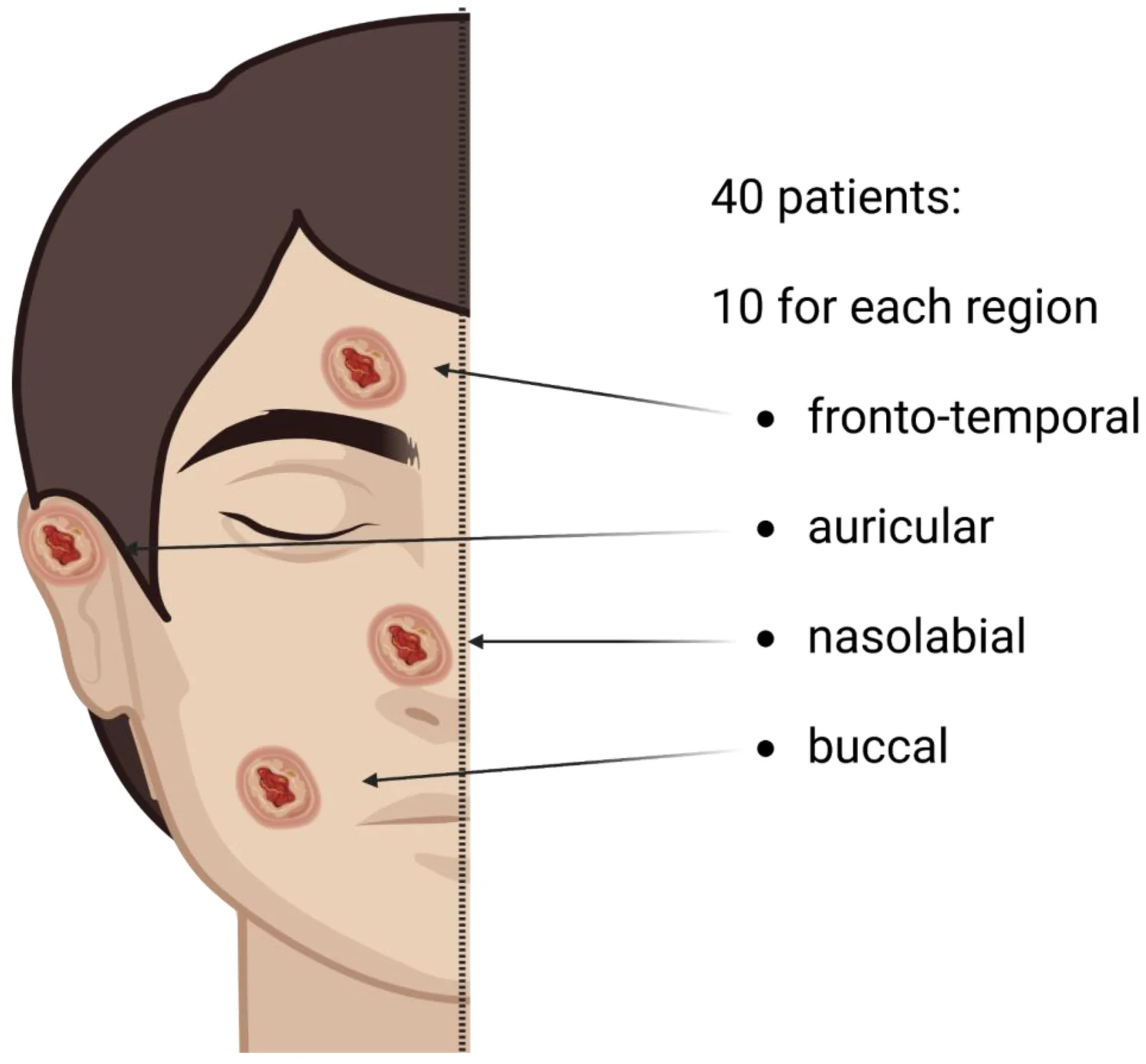
Tumor localization groups, created with BioRender.com.

The study cohort consisted of 24 male and 16 female patients, aged 45–96 years, who underwent surgery for cSCC of the facial skin. Patients were divided into four localization groups, consisting of ten patients each. Patient selection followed an intentional, stratified sampling approach to ensure equal group sizes (*n* = 10 per localization category); consecutive sampling was not applied. Exclusion criteria are stated below; A STROBE-compliant patient flow diagram is provided in the Supplementary File (Figure S1). Tumor localization per group is shown in Figure 1. According to Ishii et al., the central triangle, which consists of the nose, mouth, and eyes, has been summarized in one group as the representation of the central triangle [31]. The exclusion criteria were locoregional lymph node or distant metastasis to rule out influencing factors that are not associated with the local reconstructive treatment. Tumor-specific parameters were assessed via the histopathological results and using the eighth TNM classification according to the WHO.

All patients were evaluated before and 3 months after last surgery. Preoperative health-related quality-of-life data were collected retrospectively at the postoperative visit, introducing potential recall bias. HR-QoL was assessed using the Skindex-29, which includes 29 questions that measure physical, emotional, social, and functional well-being. Each item is scored on a 5-point scale from 0 (never) to 100 (all the time). Scores are calculated by taking the mean of the relevant items for each domain and an overall score. Higher scores indicate greater impairment of health-related quality of life [32,33]. The questionnaire was administered by the same physician, and participants were instructed to respond to each item based on their experiences over the preceding seven days. After answering the questionnaire, categorization of the recorded scores has been performed due to Nijsten et al. for a better interpretation of the values (Table 1) [34].

Demographic and clinical data, including age, sex, tumor stage, localization, and surgical technique, were collected from medical records (Table 2).

### 2.4. Statistical Analysis

Descriptive statistics were used to summarize the demographic and clinical characteristics of the study population. X^2^ Test and Wilcoxon Test were performed to determine whether there were significant changes from pre-to post-intervention among the analyzed variables. Changes in HR-QoL scores over time were analyzed using repeated-measures analysis of variance (ANOVA). A *p*-value of <0.05 was considered statistically significant; *p*-value correction due to multiple testing has been performed as <0.001 was considered statistically significant in the following. All statistical analyses were performed using SPSS version 27 (IBM Corp., Armonk, NY, USA).

## 3. Results

**Figure 2:**
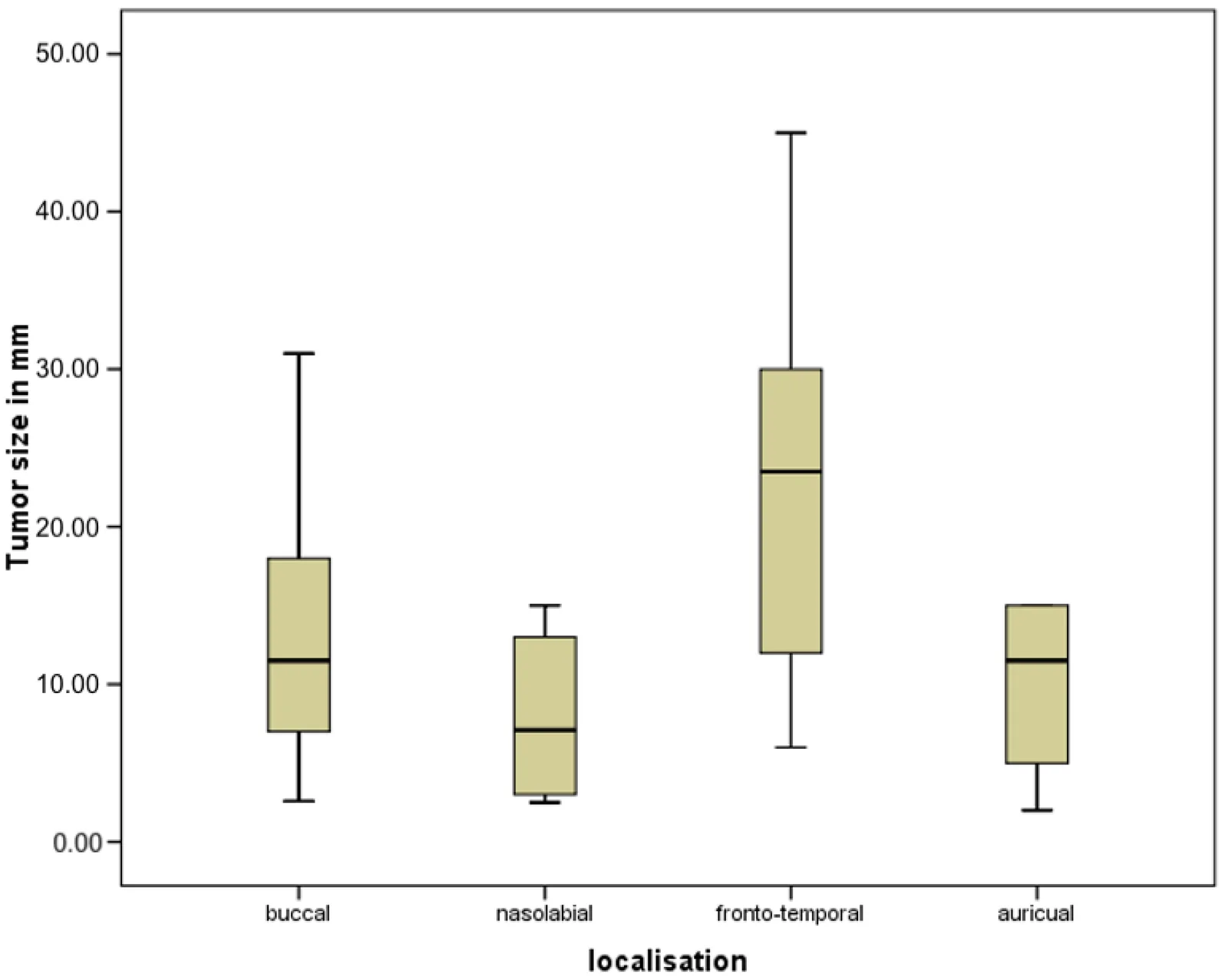
Boxplot of histopathological cSCC size in relation to localization in millimeters. Mean for evaluated groups: buccal = 13.3 mm, nasolabial = 7.83 mm, fronto-temporal = 23.54 mm, auricular = 9.9 mm.

The study included 40 patients (24 men and 16 women). The mean age was 78.5 years (SD ± 11.4). Tumor size ranged from 2 to 45 mm, with a mean of 13.7 mm among all four localization groups. No recurrences or complications during the follow-up period of three months could be observed. Preoperative domains in overall, emotions, symptoms, and function ranged from 1 to 4 in all groups, with a mean of 2.15 overall, 2.38 in emotions, 2.38 in symptoms, and 1.35 in function. Postoperative domains also ranged from 1 to 4 in all domains, except function, which ranged from 1 to 3. Mean values were 1.48 overall, 1.15 in emotions, 1.55 in symptoms and 1.13 in function. No significant differences could be found regarding age or gender in cSCC localization, but in cSCC size and localization. Fronto-temporal tumor sizes were significantly larger than the other localizations (*p* = 0.005) (Figure 2).

No correlation between surgical technique and localization, sex, age, or clinical presentation was found. However, there were tendencies to a correlation (*p* = 0.017) between surgical technique and histopathological cSCC size.

The histopathological tumor classification according to the eighth WHO classification was assessed. Tumor sizes ranged from T1 in 77.5% to T2 in 10% and T3 in 12.5% of the included patients, with no lymph node or distant metastasis. All surgical interventions were executed under sterile conditions, with local anesthesia administered in 25% of the cases and general anesthesia applied in 75% of the cases. Resection and primary wound closure were performed in a single-stage approach in 27.5% of the cases. In 72.5% of the cases, the surgical procedure was carried out in a two-stage manner, with 57.5% undergoing local flap reconstruction, 7.5% receiving a full-thickness skin graft, and 5% undergoing pedicled distant flap reconstruction. One wound was left for secondary healing. In all cases, re-resection was not necessary.

Evaluating the preoperative values, no significant differences were found in sex, age, tumor size, or localization.

Focusing on HR-QoL, significantly lower post- than preoperative scores were found in the overall (*p* = 0.00), emotions (*p* = 0.00), and symptoms (*p* = 0.00) domains, but not concerning functions (*p* = 0.167).

Paired-sample *t*-tests were performed to compare the continuous preoperative and postoperative Skindex-29 scores. Emotion scores decreased significantly from a preoperative mean of 26.00 (SD = 21.01; SE = 3.32) to a postoperative mean of 2.88 (SD = 11.49; SE = 1.82), with a mean difference of 23.13 (SD = 20.89; SE = 3.30; 95% CI: 16.44–29.81; *p* < 0.001). Symptom scores also decreased significantly from 15.45 (SD = 15.44; SE = 2.44) to 4.73 (SD = 7.77; SE = 1.23), corresponding to a mean difference of 10.71 (SD = 16.22; SE = 2.56; 95% CI: 5.53–15.90; *p* < 0.001). Function scores decreased from 5.10 (SD = 13.64; SE = 2.16) to 0.94 (SD = 3.83; SE = 0.61); however, the mean difference of 4.17 was not statistically significant (SD = 14.42; SE = 2.28; 95% CI: −0.44–8.78; *p* = 0.075). The overall Skindex-29 score decreased from 15.52 (SD = 13.33; SE = 2.11) preoperatively to 7.41 (SD = 17.59; SE = 2.78) postoperatively, with a mean difference of 8.11 (SD = 20.11; SE = 3.18; 95% CI: 1.68–14.54; *p* = 0.015).

**Figure 3:**
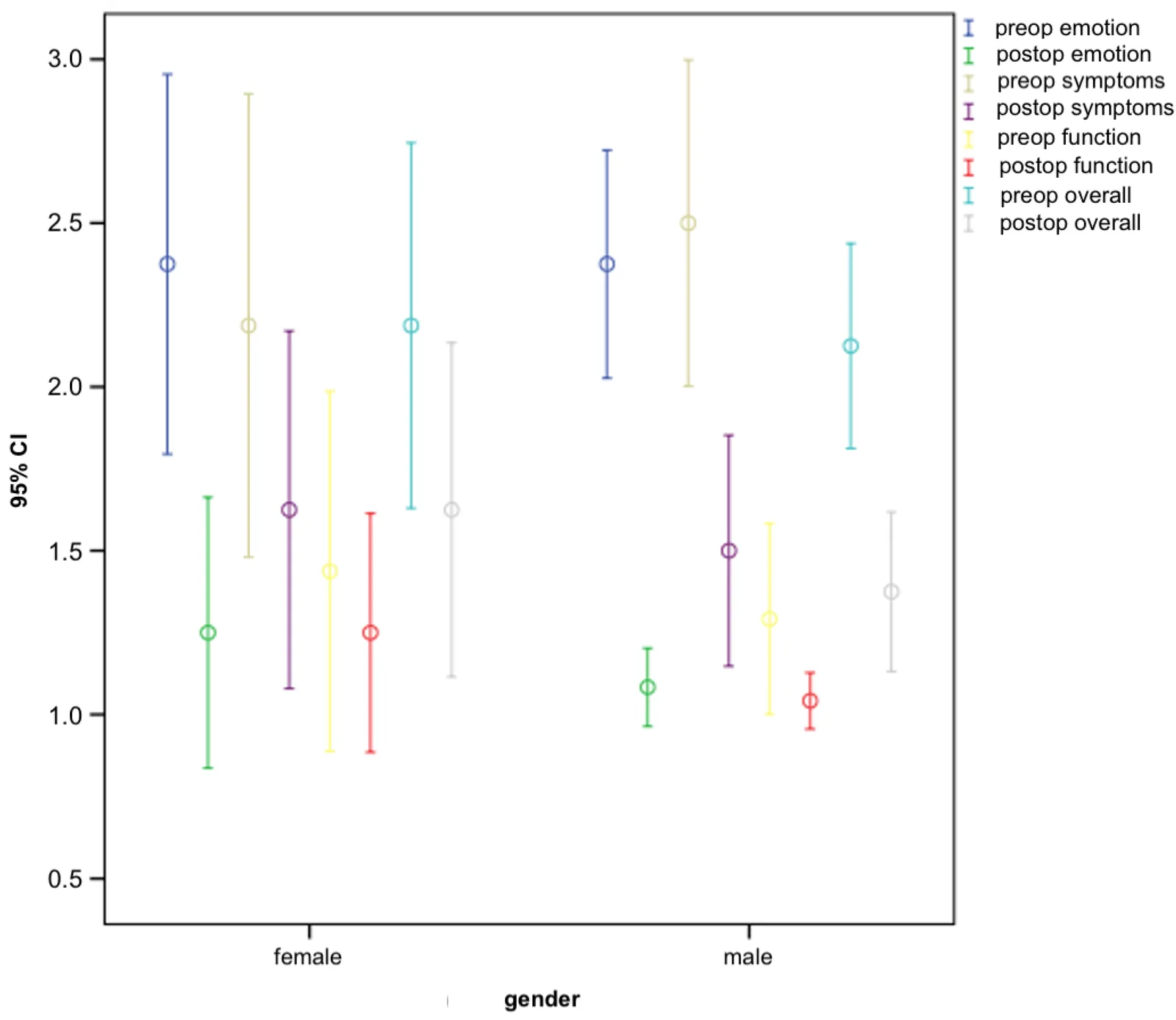
Pre- and postoperative HR-QoL domains divided by sex; *y*-axis showing skindex response categorization according to Nijsten et al. [34].

Evaluating the influence of sex on the postoperative changes in HR-QoL, women showed tendencies, due to *p*-value-correction, to improvement only in the “emotion” (*p* = 0.003) and “overall” categories (*p* = 0.046), while men exhibited significant improvement in emotions (*p* = 0.00), and tendencies in the symptoms (*p* = 0.003), and overall categories (*p* = 0.001). The values for “function” did not change in either gender (Figure 3).

To assess the impact of age on pre- and postoperative HR-QoL, patients were categorized into two age groups: younger than 80 and older than 80 at the time of surgery.

While emotions, symptoms, and overall HR-QoL improved significantly across all age groups, the changes in function, however, were not statistically significant.

Regarding “tumor localization”, improvements could be recorded in emotions in all regions. For “symptoms”, improvements were observed only in buccal and auricular tumor sites, while no changes could be found for “function” in any region. To assess tumor size, three groups were defined: <6 mm, 6–15 mm, and >15 mm, aiming for a normal distribution. Due to the broad size range within TNM T-stages, this resulted in a subdivision of various T-stages: the <6 mm category contained 8 T1 and 2 T2 tumors, the 6–15 mm group consisted exclusively of T1 tumors, and the >15 mm category comprised 3 T1, 2 T2, and 5 T3 tumors. Significant improvements across all tumor sizes were observed only in the “emotion” domain. The “function” domain showed no change. For “symptoms” and overall health-related quality of life, significant improvement was only seen in tumors measuring 6–15 mm. No statistically significant differences in HR-QoL outcomes were observed between surgical techniques; however, subgroup sizes were small, limiting the power to detect potential differences.

## 4. Discussion

This study evaluated pre- and postoperative HR-QoL in cSCC patients undergoing primary surgery. With a mean age of 78.5 years, the cohort aligns with the known incidence peak of the disease, supporting its representativeness [35]. Because AJCC-8 T-staging encompasses broad size intervals—particularly within the T1 category—it does not adequately reflect the clinically meaningful differences between small facial tumors. Our findings emphasize this limitation: TNM staging showed no association with patient-perceived HR-QoL, whereas a more detailed size-based classification offered clearer insight into postoperative HR-QoL changes [36].

Facial T1 cSCC typically ranges from 0.5 to 2.0 cm, and even small increases in diameter may necessitate wider excisions, more complex reconstruction, and potentially more noticeable scarring [37]. These subtle variations in tumor size have important consequences for cosmetic and functional outcomes, particularly in aesthetically sensitive and highly mobile regions of the face. For this reason, we applied refined size categories to more accurately evaluate how tumor extent relates to HR-QoL after surgery.

We also grouped tumors by anatomical region to reflect the heterogeneity of the face. Different regions impose distinct reconstructive challenges, functional demands, and aesthetic expectations. Following Ishii et al., the central facial triangle—comprising the eyes, nose, and mouth—was treated as a unified aesthetic unit because of its disproportionate influence on facial expression, communication, and social perception [31]. This approach enabled a more sensitive assessment to better detect potential HR-QoL differences arising from surgery in these highly expressive and functionally critical zones.

Skindex-29 was selected as the HR-QoL instrument because it captures symptoms, emotional burden, and scar-related concerns—factors that are particularly relevant in facial surgery—and has been successfully used in previous cSCC studies [29,38]. Its sensitivity to both physical and psychosocial aspects of postoperative recovery made it especially suitable for our patient population.

Postoperatively, we observed significant improvements in overall HR-QoL, symptoms, and emotional well-being. The most pronounced improvements were observed in tumors measuring 6–15 mm. This finding may reflect a balance between preoperative psychological burden and reconstructive feasibility; however, given the exploratory nature of subgroup analyses, this observation should be considered hypothesis-generating. Preoperative HR-QoL scores were already only mildly impaired, yet improved further after surgery, consistent with findings from Chren et al. in a larger cSCC cohort [29].

Baseline HR-QoL did not differ across sex, age, tumor size, or location. Postoperatively, gender differences appeared but remained modest: women showed greater emotional improvement, whereas men demonstrated broader improvements across symptoms and overall HR-QoL. These patterns reflect known gender-specific coping strategies and emotional processing but do not represent large clinical effects [39,40,41,42,43,44,45].

All age groups experienced significant postoperative HR-QoL benefits. This contrasts with findings from Leus et al., who reported no substantial HR-QoL change in frail patients using a different HR-QoL instrument [24]. Because the Skindex-29 includes a broader set of symptom and emotional domains, it may better capture improvements relevant to facial reconstruction.

Our results are consistent with prior evidence from Chren et al., demonstrating HR-QoL improvements after surgical excision and Mohs surgery but not after electrodessication and curettage [29]. Even in elderly patients, surgical treatment resulted in clear HR-QoL benefits, suggesting that age alone should not be considered a contraindication when reconstructive outcomes are expected to be favorable [23,24].

In summary, postoperative HR-QoL in facial cSCC was influenced by tumor size, localization, and sex. Within this predominantly early-stage cohort, AJCC-8 T-classification was not associated with HR-QoL outcomes. This finding should be interpreted cautiously given the limited representation of higher tumor stages. These findings suggest that more refined size-based classifications could improve clinical decision-making, enhance preoperative counseling, and better predict patient-centered outcomes.

### Limitations

This study had several limitations. First, it was conducted with a limited sample size and may not be generalizable to other populations. Second, the study did not include a control group, and the observed changes in HR-QoL may have been influenced by factors other than surgery. Third, the study relied on self-reported HR-QoL data, which may be subject to recall and social desirability bias. Patient related outcomes are by nature prone to subjectivity.

A major limitation of this study is the retrospective assessment of preoperative health-related quality of life, which may have introduced recall bias and potentially influenced the magnitude of perceived postoperative improvement. Furthermore, the follow-up period of three months is relatively short and may not fully capture long-term scar maturation, functional adaptation, and psychosocial adjustment following facial reconstruction.

Furthermore, the present study exclusively utilized Skindex-29, a disease-specific health-related quality of life instrument. The inclusion of a validated generic HR-QoL instrument, such as the WHOQOL-Bref, in future studies would allow disease-specific HR-QoL findings to be contextualized within patients’ broader overall well-being and could facilitate the evaluation of the impact of treatment on caregivers and life-partners, as has been proposed in the literature.

Despite these limitations, the study provides valuable insights into patient-reported outcomes and highlights meaningful changes in health-related quality of life following the intervention. While the findings should be interpreted with caution due to the methodological constraints, they offer useful preliminary evidence regarding the procedure’s perceived benefits. Especially in contexts where randomized controlled trials are difficult to conduct, such observational data can serve as an important complement to the existing body of evidence.

## 5. Conclusions

Surgical treatment significantly improves HR-QoL, particularly for medium-sized tumors (6–15 mm). However, TNM staging does not fully predict patient satisfaction. Gender may influence postoperative outcomes, emphasizing the need for individualized patient counseling. Further studies with larger cohorts are required to confirm these findings.

## Figures and Tables

**Table 1:** Categorization of obtained scores by Skindex-29 according to Nijsten et al. [34].

Skindex-29 Categories	Total	Emotion	Symptoms	Function
very little (1)	<5	<5	<3	<3
mild (2)	6–17	6–24	4–10	4–10
moderate (3)	18–36	25–49	11–25	11–32
severe (4)	>37	>50	26–49	>33
extremely severe (5)	-	-	>50	

**Table 2:** Demographic and clinical data, including age, sex, tumor stage, location, and surgical technique. Gender: m = male, f = female; localization: b = buccal, n = nasolabial, f = fronto-temporal, a = auricular; defect closure: L = local flap, P = primary closure, F = full-thickness skin graft, D = pedicled distant flap, S = secondary healing.

Name/ID	Gender	Age at Time of Surgery	Localization	Tumor Size in mm	Defect Closure
1	f	51	b	2.6	L
2	f	77	b	22	L
3	m	75	b	15	P
4	f	74	b	18	L
5	m	82	b	7.7	P
6	f	85	b	7	L
7	f	85	b	7	L
8	m	87	b	31	P
9	m	86	b	8	L
10	m	61	b	15	L
11	f	92	n	10	P
12	m	65	n	2.6	P
13	m	93	n	9.2	L
14	f	76	n	4	D
15	f	49	n	14	L
16	f	45	n	5	L
17	f	81	n	15	L
18	m	79	n	13	L
19	m	82	n	3	P
20	f	92	n	2.5	L
21	m	68	f	25	F
22	m	74	f	8	L
23	f	81	f	22	F
24	m	79	f	12	L
25	f	84	f	6	P
26	m	88	f	30	L
27	f	71	f	45	F
28	m	87	f	42	D
29	f	88	f	30	L
30	f	80	f	15.4	P
31	m	76	a	9	L
32	m	78	a	8	S
33	m	76	a	14	L
34	m	83	a	5	P
35	m	83	a	2	P
36	m	80	a	15	L
37	m	88	a	2	L
38	m	88	a	14	L
39	m	94	a	15	L
40	m	76	a	15	P

## Data Availability

The datasets used and/or analyzed during the current study are available from the corresponding author on reasonable request.

## Supplementary Materials

The following supporting information can be downloaded at: https://www.mdpi.com/article/10.3390/cmtr19030037/s1, Figure S1: STROBE Flow Diagram—Patient Inclusion and Follow-Up.
